# Alterations in neuronal activity in basal ganglia-thalamocortical circuits in the parkinsonian state

**DOI:** 10.3389/fnana.2015.00005

**Published:** 2015-02-05

**Authors:** Adriana Galvan, Annaelle Devergnas, Thomas Wichmann

**Affiliations:** ^1^Yerkes National Primate Research Center, Emory UniversityAtlanta, GA, USA; ^2^Department of Neurology, School of Medicine, Emory UniversityAtlanta, GA, USA; ^3^Udall Center of Excellence for Parkinson’s Disease Research, Emory UniversityAtlanta, GA, USA

**Keywords:** Parkinson’s disease, parkinsonism, basal ganglia, electrophysiology, extracellular recording, LFP, animal models

## Abstract

In patients with Parkinson’s disease and in animal models of this disorder, neurons in the basal ganglia and related regions in thalamus and cortex show changes that can be recorded by using electrophysiologic single-cell recording techniques, including altered firing rates and patterns, pathologic oscillatory activity and increased inter-neuronal synchronization. In addition, changes in synaptic potentials or in the joint spiking activities of populations of neurons can be monitored as alterations in local field potentials (LFPs), electroencephalograms (EEGs) or electrocorticograms (ECoGs). Most of the mentioned electrophysiologic changes are probably related to the degeneration of diencephalic dopaminergic neurons, leading to dopamine loss in the striatum and other basal ganglia nuclei, although degeneration of non-dopaminergic cell groups may also have a role. The altered electrical activity of the basal ganglia and associated nuclei may contribute to some of the motor signs of the disease. We here review the current knowledge of the electrophysiologic changes at the single cell level, the level of local populations of neural elements, and the level of the entire basal ganglia-thalamocortical network in parkinsonism, and discuss the possible use of this information to optimize treatment approaches to Parkinson’s disease, such as deep brain stimulation (DBS) therapy.

## Introduction

Parkinson’s disease (PD) is primarily characterized by movement deficits, but encompasses also many non-motor problems. The term “parkinsonism” refers to a characteristic constellation of motor impairments that are associated with PD, including decreased and slow movement (akinesia and bradykinesia), muscular rigidity, gait instability, and tremor at rest. The presence of bradykinesia and at least one other signs is required for the formal diagnosis of PD (Hughes et al., 1992).

Parkinsonism results, in large part, from the degeneration of dopaminergic neurons in the pars compacta of the substantia nigra (SNc; Bernheimer et al., 1973), and the resulting reduction in the levels of dopamine in the striatum, the main synaptic target of SNc axons (Hornykiewicz and Kish, 1987). The loss of dopamine is correlated with profound changes in the activity of neurons in the basal ganglia-thalamocortical circuits. In this review, we summarize the current knowledge of some of the electrophysiological changes that occur in the basal ganglia and related thalamic and cortical areas in parkinsonism. We focus on results obtained with extracellular recordings in parkinsonian animals or in human PD patients. Studies of functional abnormalities can also be conducted using non-electrophysiological methods (for instance, by using markers of metabolism or imaging techniques). The results of these studies are not covered here in any detail, but have been discussed in other publications (e.g., Galvan and Wichmann, 2008; Lindenbach and Bishop, 2013).

## Circuit anatomy and pathology of Parkinson’s disease

### Functional anatomy of the basal ganglia-thalamocortical circuits

**Figure 1:**
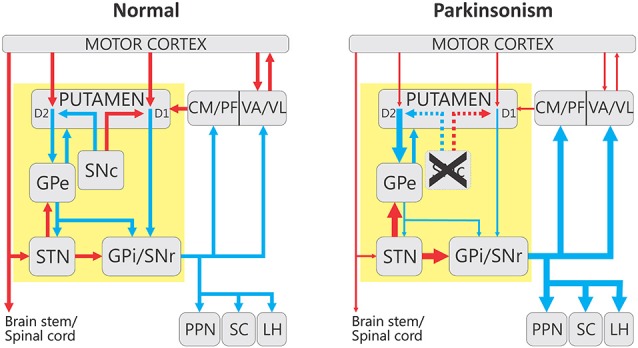
**Firing rate changes in the basal ganglia-thalamo-cortical motor circuit in parkinsonism**. The yellow box indicates the interconnected basal ganglia nuclei that receive extrinsic input from the cortical and thalamic regions. For simplicity some connections have been omitted from this diagram. The left panel indicates the circuits in the “Normal” state, and the right shows the overall changes in activity that have been associated with parkinsonism. Blue and red arrows indicate inhibitory and excitatory connections, respectively. The thickness of the arrows corresponds to their presumed activity. Abbreviations: CM, centromedian nucleus of thalamus; D1 and D2, dopamine receptor subtypes; GPe, external segment of the globus pallidus; GPi, internal segment of the globus pallidus; LH, lateral habenula; PF, parafascicular nucleus of the thalamus; PPN, pedunculopontine nucleus; SC, superior colliculus; SNc, substantia nigra pars compacta; SNr, substantia nigra pars reticulata; STN, subthalamic nucleus; VA, ventral anterior nucleus of thalamus; VL, ventrolateral nucleus of thalamus.

**Figure 2:**
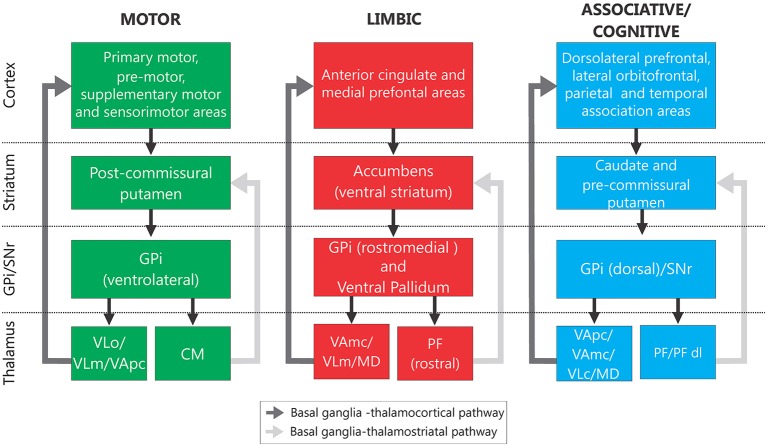
**Segregated basal ganglia—thalamocortical and basal ganglia—thalamostriatal functional loops**. Information related to each functional modality is processed through segregated regions of the cerebral cortex, the basal ganglia and thalamic nuclei. Abbreviations: See Figure 1 and MD, mediodorsal nucleus; PFdl, dorsolateral extension of the PF; VAmc, part magnocellular of VA; VApc, part parvocellular of VA; VLc, caudal part of VL; VLd, dorsal VL; VLo, pars oralis of VL; VLm, medial part of VL (based on Alexander et al., 1986, 1990; Middleton and Strick, 2000).

The basal ganglia (Figure 1, “Normal”) consist of the neostriatum (caudate nucleus and putamen), the external and internal pallidal segments (GPe, GPi), the subthalamic nucleus (STN), the substantia nigra pars reticulata (SNr), and the SNc. These structures are part of larger functional and anatomical parallel circuits that also include areas of the frontal cortex and of the ventral thalamus. Depending on the function of the frontal cortical area of origin, these basal ganglia-thalamo-cortical circuits are designated as “motor”, “associative/cognitive” and “limbic” (Figure 2, Alexander et al., 1986, 1990; Middleton and Strick, 2000).

The general anatomical arrangement of the basal ganglia is similar across these circuits. Glutamatergic excitatory cortical input reaches the basal ganglia via the striatum and STN, and, to a lesser extent, via the thalamus. The information is then transferred to the output nuclei of the basal ganglia, the GPi and SNr. The projections from the striatum to the output nuclei are divided into the monosynaptic “direct” pathway, and the “indirect” pathway, a polysynaptic projection that traverses GPe and STN before reaching the output nuclei. The STN is another major entry station for extrinsic cortical information into the basal ganglia. The cortico-subthalamic route, together with its continuation to GPi/SNr has been termed the “hyperdirect” pathway because information flowing along this projection reaches the basal ganglia output structures with a shorter delay than information transmitted along the direct and indirect corticostriatofugal systems (Nambu et al., 2002).

GPi and SNr neurons project to the thalamus, the lateral habenula, and brainstem structures, including the superior colliculus, and the pedunculopontine nucleus. There are also thalamic projections to the basal ganglia which reach primarily the striatum, with lesser projections to GPe and STN (Sadikot and Rymar, 2009; Smith et al., 2014a).

The obvious topographic separation of motor and non-motor functions at the cortical level is maintained throughout the subcortical course of the cortico-basal ganglia-thalamocortical circuits(Alexander et al., 1986). As mentioned below, abnormal activity patterns in the *motor* circuit are correlated with the appearance of parkinsonism. This circuit originates in somatosensory, motor, and premotor cortices, which innervate the post-commissural putamen, preserving a somatotopic organization. This area of the striatum projects, in turn, to motor regions of GPe, STN, GPi and SNr. The output nuclei send their axons to motor regions of thalamus, specifically to the ventral anterior and ventrolateral nuclei of the thalamus (VA/VL), which project back to motor regions of cortex. The GPi and SNr also send collaterals to the intralaminar centromedian and parafascicular thalamic nuclei (CM/PF) which, in turn, send glutamatergic efferents to the striatum (Sadikot and Rymar, 2009; Smith et al., 2014a). Of these, the CM nucleus receives movement-related output from GPi, and projects to the movement-related area of the striatum (the putamen), while PF receives largely non-motor-related inputs from the basal ganglia, and projects to the non-motor caudate nucleus.

While most basal ganglia structures are composed of either GABAergic (GPe, GPi and SNr) (Oertel and Mugnaini, 1984; Smith et al., 1987; Ilinsky et al., 1997) or glutamatergic (STN) projection neurons (Smith and Parent, 1988), the striatum contains a large population of GABAergic medium-spiny projection neurons (MSNs), and a smaller, but functionally important, proportion of interneurons, including cholinergic and various types of GABAergic interneurons (Kawaguchi, 1993; Tepper and Bolam, 2004). Besides these intrinsic sources of GABA, the striatum also receives GABAergic input from a subset of GPe neurons (Bevan et al., 1998; Sato et al., 2000; Mallet et al., 2012; Mastro et al., 2014).

In addition, the striatum receives a dense dopaminergic innervation from neurons in the SNc. Dopamine is a critical neuromodulator of striatal activity, acting both presynaptically and postsynaptically. We will describe its (proposed) actions in some detail here, because knowledge of the effects of the physiologic dopamine will facilitate an understanding of the effects of dopamine loss in PD.

At the presynaptic level, dopamine acts to decrease the release of glutamate from the terminals of cortical or thalamic projections, as well as the release of GABA from interneurons (reviewed in Tritsch and Sabatini, 2012). Postsynaptically, dopamine modulates the excitability and responsiveness of MSNs and striatal interneurons (Tritsch and Sabatini, 2012).

Dopamine D1-like and D2-like receptors are differentially expressed on the direct and indirect pathway MSNs, respectively (Figure 1, Gerfen et al., 1990). Because these receptor families are coupled to different second messenger system (through Ga_s_ or Ga_olf_ for D1-like receptors, and Ga_i_ or Ga_o_ for D2-like receptors, Tritsch and Sabatini, 2012), activation of D1 receptors increase the excitability of direct pathway MSNs, and facilitates long-term potentiation at glutamatergic synapses terminating on them, while activation of D2 receptors decreases the excitability of indirect pathway MSNs, and promotes long-term depression at synapses of the cortical projections terminating on their dendrites (Surmeier et al., 2011, 2014; Tritsch and Sabatini, 2012).

By increasing activity along the direct pathway, dopamine is thought to lead to an inhibition of the output nuclei of the basal ganglia. In contrast, by suppressing the activity of indirect pathway MSNs, dopamine facilitates GPe-STN transmission, thus increasing the inhibition of STN neurons (downstream from GPe) and their projections to GPi and SNr. The combination of these effects is that GPi and SNr activity is reduced by dopamine release in the striatum, leading to a reduction of the inhibition of thalamocortical projection neurons that receive the input from these nuclei. The differential dopaminergic modulation of the strength of synapses on direct and indirect pathway MSNs has been proposed to be relevant for the formation of reward-based procedural memory. The general functional model of the interactions between direct and indirect pathway in the regulation of motor activity was proposed decades ago (Figure 1, Albin et al., 1989; DeLong, 1990), and has received strong experimental support in the last few years (Kravitz et al., 2010, 2012; Cui et al., 2013; Freeze et al., 2013; Sano et al., 2013).

### Loss of dopamine and plastic changes in the basal ganglia in PD

In PD, the dopaminergic nigrostriatal pathway progressively degenerates. The dopaminergic projections to the sensorimotor striatum are affected more strongly than those to the associative and limbic striatal regions, contributing to the preponderance of movement problems in PD (Kish et al., 1988; Brooks et al., 1990). The loss of striatal dopamine is associated with morphological (non-dopaminergic) changes throughout the basal ganglia, including a reduction in the density of dendritic spines of MSNs in the striatum and alterations in intrastriatal and pallido-subthalamic connectivity, as we discuss below.

Although the loss of dopaminergic SNc cells remains the best known pathological feature of PD, it is clear that other types of neurons also degenerate, including neurons in the serotonergic raphe nuclei, the noradrenergic locus coeruleus, the olfactory tubercle, the intralaminar nuclei of the thalamus, and regions of the cerebral cortex and the peripheral nervous system (Braak and Braak, 2000; Braak et al., 2006; Sulzer and Surmeier, 2013). It is still not well understood how these changes in the brain stem, which occur relatively early in the disease, contribute to the motor and non-motor manifestations of parkinsonism.

## Techniques used to study pathophysiologic changes in parkinsonism

### Analytical approaches to extracellular recordings and definition of terms

Most electrophysiologic *in vivo* recordings utilize extracellular recording methods, thus reflecting electrical potentials at the sites of electrodes that are positioned in the extracellular space, at some distance from the sources of the electrical activity, i.e., neuronal cell bodies or axons. The principal contributors to the recorded potentials are neuronal events in which ions are moving, i.e., action potentials and synaptic currents. The former can be detected as spikes in extracellular recordings, while the latter are recordable (at least in principle) as low-frequency fluctuations of recorded potentials.

### Analysis of single cell discharge (action potentials)

**Figure 3:**
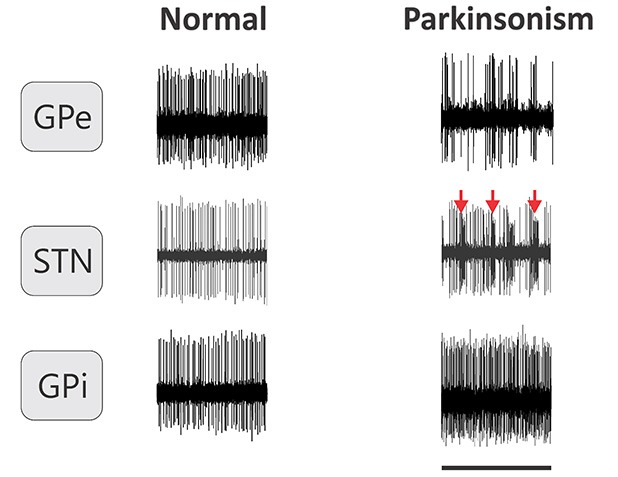
**Examples of extracellular recordings of individual neurons in the GPe, GPi and STN of monkeys in normal conditions and after MPTP treatment**. Arrows indicate examples of bursts in an STN neuron. Horizontal line represents 1 s.

The shape of individual action potentials is heavily dependent on the distance of the recording site from the current-generating source, and the geometric arrangement of the two. Figure 3 shows examples of traces obtained during extracellular recordings of single neurons in the basal ganglia of monkeys. The analysis of neuronal action potential activity focuses therefore mostly on the timing of these potentials, and is conventionally expressed in the form of inter-spike intervals (ISIs).

The most basic parameters that can be provided by an analysis of ISIs from an individual neuron are the static firing rate, along with measurements of the variability of discharge, such as a calculation of the coefficient of variation of ISIs, and assessments of the distribution of the ISIs (e.g., Kaneoke and Vitek, 1996).

Other analyses examine the preponderance of specific firing patterns, specifically bursts of action potentials. Such bursts are intuitively defined as sudden episodic unexpected accelerations in discharge rates, and analyzed by a variety of formal burst detection algorithms (Grace and Bunney, 1984; Legendy and Salcman, 1985; Aldridge and Gilman, 1991; Wichmann and Soares, 2006; Shimo and Wichmann, 2009).

Another set of features that can be conveniently studied is oscillatory activity. When applied to recordings of spiking activity of single neurons, the term “oscillation” is used to describe periodic fluctuations in the instantaneous firing rate of the neuron. Oscillatory activity can be most easily analyzed by frequency-domain methods, including global or time-resolved power spectral analyses of a neuron’s discharge. Oscillatory activity is often grouped into larger frequency bands, based on conventions from the EEG literature, where rhythmic activity within specific frequency ranges is found in certain distribution patterns in scalp recordings, or is thought to have biological significance. Such frequency bands include the delta band (below 4 Hz), the theta band (4–8 Hz), the alpha band (8–13 Hz), the beta band (13–30 Hz) and the gamma band (above 30 Hz). The use of these band designations has led to much confusion in the field. One problem is that splitting the frequency spectrum in the EEG-based way may not be useful for other types of brain recordings. Many researchers therefore break the larger frequency ranges into smaller ones (such as the “low-beta” and “high-beta” band), or use the terminology loosely (for instance, using the term “beta band” for oscillations that encompass parts of the classical alpha- and beta bands). Another reason that argues against the unquestioning use of the traditional band descriptions is that bands of specific biological or pathological significance may differ between species, so that beta band oscillations in one species may be equivalent to oscillations in other bands in another (discussed in ref. Stein and Bar-Gad, 2013).

Oscillations of firing can occur with or without bursting (Wichmann and Soares, 2006; Wichmann and Dostrovsky, 2011). When slow oscillations occur in the form of periodically recurring bursts, this may represent the coupling of two different oscillatory phenomena (the amplitude of high frequency oscillations within the bursts is modulated by the phase of the low oscillation represented by the grouping of bursts), as is discussed to also be a prominent feature of cortical field potential recordings in parkinsonian patients (de Hemptinne et al., 2013).

Time domain measures (such as firing rates) and frequency domain measures (such as power spectra) are not sensitive to “nonlinear” features of discharge. The analysis of such non-linear features remains currently in its infancy (e.g., ref. Rodríguez et al., 2003; Darbin et al., 2006; Dorval et al., 2008), and is an interesting area of exploration.

Another level of analysis of single cell discharge is that of examination of the relatedness of activity patterns of multiple neurons. A commonly used time-domain technique is to compute the cross-correlation, a measure that examines the likelihood of firing in one neuron at pre-determined time intervals before or after another cell’s firing. If oscillatory activity is the focus of the analysis, frequency-domain techniques (such as cross-spectra or coherence measurements) can also be applied.

### Analysis of electrical activity of neuronal populations

Field potential recordings (including recordings of local field potentials (LFPs), electrocorticograms (ECoGs), and electroencephalograms (EEGs)) offer a rich source of information. It is generally accepted that LFPs are generated by transmembrane flow of current, and thus reflect synaptic activity within a population of neurons in an area several hundred micrometers in diameter around the electrode (Buzsáki et al., 2012) (but see Kajikawa and Schroeder, 2011). While relatively easy to record, the interpretation of these data is not straightforward. Field potential recordings are most often analyzed using frequency domain analysis methods, comparing the spectral content of the signals in different behavioral states. Another use of these signals is to analyze the presence of stimulation- or event related potentials, i.e., synaptic potentials related to electrical stimulation of the tissue, or potentials that precede or follow a behavioral event. Individual evoked potentials are often not visible, so that averaging techniques have to be used to extract stereotypically recurring waveforms.

Field potential signals recorded simultaneously at different locations within the brain can also be used for cross-spectral or coherence analyses which can provide information on the coupling of activity of brain regions. Phase shifts in such studies have been used to identify causality relationships, or to determine the direction of information flow within the network under study.

### Extracellular recordings in humans

In recent years, functional neurosurgical interventions, such as lesioning or DBS procedures, have been increasingly used to treat patients with PD. The placement of lesions or DBS electrodes in the brain is often done guided by intraoperative electrophysiologic recordings. Microelectrode recordings from these surgeries have been extensively used to analyze the activity of the basal ganglia and related cortical and thalamic structures in PD patients. In addition, researchers have also used the implanted DBS electrodes as (macro-) electrodes to record LFPs intraoperatively, or in the immediate postoperative period.

Recordings in humans suffer from the unavoidable limitation that there are no available recordings in healthy subjects to use as controls. In some cases, recordings from patients with other basal ganglia-related neurological conditions (e.g., essential tremor, dystonia) are used for comparison instead.

### Animal models of Parkinson’s disease

Animal models of PD can be divided into several categories, including models that seek to replicate some of the genetic abnormalities that can lead to parkinsonism (Dawson et al., 2010; Blesa et al., 2012); models that attempt to mimic the spread of pathology (such as the intraparenchymal infusion of alpha-synuclein oligomers, e.g., Bezard et al., 2013), models that use pharmacological compounds to acutely deplete dopamine or to antagonize dopaminergic transmission (Bezard and Przedborski, 2011), and models that utilize neurotoxins that damage dopaminergic brain neurons (Beal, 2001; Dauer and Przedborski, 2003; Blesa et al., 2012). Electrophysiologic studies have heavily relied on the toxin models, so, to a large extent, our knowledge of the “pathophysiology of the parkinsonian state” is a description of the consequences of dopamine cell loss.

The two most common toxins used to create animal models of PD are 6-hydroxydopamine (6-OHDA) and 1-methyl-4-phenyl-1,2,3,6-tetrahydropyridine (MPTP). Other toxins that have been described include pesticides and herbicides such as rotenone and paraquat (Betarbet et al., 2000; Hirsch et al., 2003; Thiruchelvam et al., 2003; Bové et al., 2005; Blesa et al., 2012). 6-OHDA was the first compound used to selectively lesion dopaminergic pathways (Ungerstedt, 1968). This toxin is injected directly in the brain parenchyma, targeting either the SNc itself, at the level of the dopaminergic terminals in the striatum or in the median forebrain bundle, which carries ascending dopaminergic projections to the forebrain (Schwarting and Huston, 1996b). Most frequently, 6-OHDA injections are done unilaterally, while the contralateral side serves as an (imperfect) control (Ungerstedt, 1968; Schwarting and Huston, 1996a,b). The toxin is effective in rats, mice, monkeys and other species, but has been most frequently used in rats. Dopamine depletion after unilateral injection of 6-OHDA results in motor asymmetry which can be easily quantified with behavioral methods (Ungerstedt and Arbuthnott, 1970; Ungerstedt, 1971; Schwarting and Huston, 1996a).

MPTP was discovered to induce dopamine loss in the striatum in the early 1980’s, when a group of drug users developed parkinsonism after accidentally self-administering the toxin (Langston et al., 1983). Shortly after this discover, MPTP was shown to produce irreversible phenotypically convincing parkinsonism in monkeys as well (Burns et al., 1983; Langston et al., 1984; Langston and Irwin, 1986), which can be alleviated by dopaminergic agonists (e.g., Nomoto et al., 1985; Nomoto and Fukuda, 1993), deep brain stimulation (DBS, e.g., Benazzouz et al., 1993; Johnson et al., 2009; Rosin et al., 2011; Vitek et al., 2012) and other antiparkinsonian treatments. In monkeys, MPTP is most commonly administered by systemic injection, resulting in bilateral parkinsonism (Przedborski et al., 2001). Daily recurring treatment with high-dose MPTP results in rapidly evolving severe parkinsonism, while less frequent chronic administration of small doses of MPTP for a prolonged period of time (for instance, weekly injections for several months) results in slowly developing parkinsonism (Albanese et al., 1993). The latter model has been useful in studying the progression of electrophysiological changes in the basal ganglia in relation to the appearance of motor deficits (Devergnas et al., 2014). Another method of applying MPTP is administration of the toxin via intracarotid infusion, resulting in hemiparkinsonism (Bankiewicz et al., 1986).

MPTP can also induce degeneration of dopaminergic neurons in mice and rats, although, much higher doses of the toxin are needed than those required for monkeys (Chiueh et al., 1984; Heikkila et al., 1984; Kopin and Markey, 1988; Giovanni et al., 1994a,b). MPTP treatment in rodents tends to have more variable and unstable behavioral outcomes, and is therefore not commonly used for pathophysiologic studies.

## Changes in neuronal activity in parkinsonism

Early studies of the electrophysiologic alterations in MPTP-treated monkeys described mostly changes in the firing rates of individual neurons after dopamine depletion (Filion, 1979; Miller and DeLong, 1987, 1988), including decreases in firing rate in GPe neurons, and increased firing in STN, GPi and SNr cells. These modifications in firing rates are accompanied by changes in firing patterns, such as an increased tendency of neurons to fire in bursts of action potentials, enhanced oscillatory (rhythmic) activity within each nucleus and among structures, and increased synchrony among neighboring neurons.

In the following sections we will describe in more detail the changes in neuronal firing at the single cell and population levels for each basal ganglia structure, as well as for the associated cortical and thalamic regions. Although each nucleus will be presented separately, it is important to keep in mind that parkinsonian-related changes are the result of network alterations, thus affecting simultaneously all elements of the basal ganglia thalamocortical circuitry. Table 1 shows a summary of the changes reported for each structure.

**Table 1:** **Electrophysiological changes reported in parkinsonism in the basal ganglia and related thalamic and cortical regions**.

	Firing rate	Bursting	Oscillations (single cell studies)	Oscillations (LFP studies)	Interneuronal synchrony
Striatum
NS	INC. (R, P) (Chen et al., 2001; Liang et al., 2008)			INC. (M, beta) (Costa et al., 2006).
MSNd	DEC. (R) (Mallet et al., 2006; Kita and Kita, 2011)
MSNi	INC. (R) (Mallet et al., 2006; Kita and Kita, 2011)
TAN			INC. (P, beta) (Raz et al., 1996)		INC. (P) (Raz et al., 1996)
GPe	DEC. (P, R, H) (Miller and DeLong, 1987; Pan and Walters, 1988; Sterio et al., 1994)	INC. (P, R, H) (Hutchison et al., 1994; Vila et al., 2000; Wichmann and Soares, 2006).	INC. (P, R, theta, alpha) (Raz et al., 2000; Magill et al., 2001)		INC. (P, R) (Nini et al., 1995; Mallet et al., 2008a)
STN	INC. (R, P, H) (Bergman et al., 1994; Hassani et al., 1996)	INC. (P, H) (Bergman et al., 1994; Steigerwald et al., 2008)	INC. (P, theta, alpha; H, beta) (Bergman et al., 1994; Levy et al., 2002b)	INC. (H, R, beta) (Priori et al., 2004; Sharott et al., 2005a) DEC. (H, gamma) (Brown et al., 2001)	Prominent (H, patients with limb tremor) Levy et al. (2002b)
GPi/SNr	INC. (P, H) (Miller and DeLong, 1987; Hutchison et al., 1994; Wichmann et al., 1999)	INC. (P, H) (Raz et al., 2000; Chan et al., 2011)	INC. (P, theta, alpha) (Raz et al., 2000; Soares et al., 2004)	INC. (P, alpha; H, beta) (Brown et al., 2001; Devergnas et al., 2014)	INC. (P, H) (Nini et al., 1995)
Thalamus (VA/VL)	Not consistent (P, H, R) (Vitek et al., 1994; Molnar et al., 2005; Pessiglione et al., 2005)	INC. (P, H) (Zirh et al., 1998; Magnin et al., 2000; Guehl et al., 2003)	INC. (P, theta, alpha) (Kammermeier et al., 2014)	INC. (H, theta) (Sarnthein and Jeanmonod, 2007)
Motor cortex
NS	DEC. (P, movement- related) (Doudet et al., 1990)	INC. (P) (Goldberg et al., 2002)		INC. (H, delta; H, P, theta; P, alpha; R, beta) (Neufeld et al., 1994; Sharott et al., 2005a; Devergnas et al., 2014) DEC. (P, gamma) (Devergnas et al., 2014)	INC. (P) (Goldberg et al., 2002)
Cortico-striatal	DEC. (R, to MSNd) (Mallet et al., 2006) UNCH. (P) (Pasquereau and Turner, 2011)	UNCH. (P) (Pasquereau and Turner, 2011)
Cortico-spinal	DEC. (P) (Pasquereau and Turner, 2011)	INC. (P) (Pasquereau and Turner, 2011)

*Limits used to define frequency bands in the columns referring to oscillations: delta, <4 Hz; theta, 4–8 Hz; alpha, 8–13 Hz; beta, 13–30 Hz; gamma >30 Hz (see Section 1.4.1). Abbreviations: NS, not specified; MSNd, medium spiny neurons of the direct pathway; MSNi medium spiny neurons of the indirect pathway; TAN, tonically active neurons; Inc, increased; Dec, decreased; Unch, unchanged; P, MPTP-treated primates); R, 6-OHDA treated rats; H, PD patients; M, DAT-KO mice (see ref. Costa et al., 2006). A few representative examples of the studies in which these changes are demonstrated are cited in the table. Other references can be found in the main text*.

### Striatum

The original “rate” model of the basal ganglia dysfunction in PD (Figure 1, “Parkinsonism”, Albin et al., 1989; DeLong, 1990), proposed that striatal MSNs that give rise to the direct or indirect pathway are differentially affected by the striatal dopamine loss in PD. While direct pathway neurons reduce their firing rate, those in the indirect pathway increase their activity. As a result, the firing in GPe neurons is reduced, which leads to disinhibition of STN neurons and, subsequently, excessive excitation of STN targets (GPi and SNr). The increased activity of GPi and SNr neurons is further reinforced by the lack of inhibition from direct pathway neurons. The final outcome of these changes is that the basal ganglia exert a greater than normal inhibition on their thalamic and brainstem targets.

Evidence in favor of increased activity of some neurons in the striatum was found in parkinsonian rats and monkeys (Chen et al., 2001; Liang et al., 2008). By using antidromic stimulation or juxtacellular labeling, studies in 6-OHDA treated rats have identified MSNs from the direct or the indirect pathway. These results indicate that, after dopaminergic depletion, direct pathway MSNs are less active, while the spontaneous firing of indirect pathway MSNs is increased (Mallet et al., 2006; Kita and Kita, 2011). The changes in firing rate in MSNs could be, at least in part, a consequence of changes in corticostriatal projections rather than an intrinsic change in activity, since cortical input to direct pathway MSNs (identified by showing antidromically mediated responses to stimulation of the SNr) show decreased spontaneous activity in 6-OHDA treated rats (Mallet et al., 2006), while inputs to neurons that did not respond antidromically to the SNr stimulation (presumed to be indirect pathway MSNs) were unchanged. It is not clear whether this finding also applies to primates, as studies in monkeys have suggested that corticostriatal projection neurons show few (if any) changes in activity in the parkinsonian state (Pasquereau and Turner, 2011). The rate model has recently received additional support from the application of optogenetic techniques in parkinsonian mice, in which selective activation of opsin-expressing direct pathway MSNs alleviated the motor deficits resulting from dopamine depletion (Kravitz et al., 2010). This study, however, does not provide direct evidence that low activity of direct pathway MSNs caused the motor deficits.

Due to the low number of interneurons in the striatum, changes in the electrical activity of these neurons in parkinsonism have been studied to a very limited extent. A specific group of cells, however, the tonically active neurons (TANs presumed to be cholinergic interneurons, Goldberg and Reynolds, 2011), has received considerable attention. These cells have a regular tonic firing at rest, a wide action potential, and a stereotypic electrophysiological response to rewarding events (Apicella, 2002). After MPTP treatment, the number of TANs showing this characteristic response to reward was drastically reduced, and a large proportion of TANs develop abnormal oscillatory activity around 16 Hz, while the already high degree of correlation between these neurons was slightly increased (Raz et al., 1996).

Few studies have analyzed parkinsonism-related changes in oscillatory activities in LFP signals in the striatum. In mice, spectral power in the delta and beta ranges was increased in LFPs recorded in the dorsal striatum during pharmacologically-induced acute akinesia, and such increases were reversed by administration of L-DOPA (Costa et al., 2006). While intrastriatal blockade of D2- and D1-receptors induce severe akinesia, only blockade of D2-receptors increases delta and beta oscillations and decreases gamma activity in the striatum (Burkhardt et al., 2009). Other studies have suggested that the effect of dopamine depletion on LFP oscillations in the striatum may be task- and learning-dependent (Lemaire et al., 2012), and that it is strongly influenced by oscillatory activity in the cortex, as shown by simultaneous recordings of striatal LFPs and EEGs (Courtemanche et al., 2003; Berke et al., 2004).

The parkinsonian state has frequently been associated with alterations in the somatotopic organization of the basal ganglia-thalamo-cortical circuits. In the striatum of parkinsonian rodents, there is a disruption of the normal organization of striatal neurons that response to sensory inputs (Cho et al., 2002). This finding may indicate that cortical-striatal inputs are abnormal (perhaps related to the morphological changes discussed below), or could reflect pathological processing of information within the striatum itself.

Several morphological changes occur in the striatum after dopaminergic depletion and may contribute to the appearance of abnormal activity patterns in this nucleus. A major alteration, first described in 6-OHDA rats and later in PD patients and MPTP-treated monkeys, is the loss of dendritic spines of MSNs (Ingham et al., 1989; Stephens et al., 2005; Zaja-Milatovic et al., 2005; Day et al., 2006; Villalba et al., 2009). Since the dendritic spines on MSNs are the main recipients of cortico-striatal (and a proportion of thalamo-striatal) afferents, it is likely that the spine loss affects glutamatergic transmission in the striatum (Smith and Villalba, 2013). As studied in MPTP-treated monkeys, the remaining spines, along with the corticostriatal and thalamostriatal terminals that contact them, have an increased volume compared to that found in normal animals, which may signify increased activity at these synapses (Villalba and Smith, 2011). Studies in 6-OHDA treated mice have also demonstrated that the dendritic arborization is reduced in both direct and indirect pathway MSNs (Fieblinger et al., 2014) (These morphological alterations are accompanied by functional changes, as demonstrated recently in *in vitro* studies, which show that after dopaminergic lesions with 6-OHDA the intrinsic and dendritic excitability of indirect pathway MSNs was lower, while the intrinsic excitability of direct pathway MSNs was increased (Fieblinger et al., 2014), compared to the normal state. These changes were interpreted as homeostatic adaptations that could contribute, in early stages of parkinsoninsm, to keep a balance activity of both striatal patwhays, but that are insufficient in later stages (Mallet et al., 2006). Such plastic changes remain to be fully explored in *in vivo* animal models of PD (Ma et al., 2012).

Other plastic changes in striatal connectivity have been described in parkinsonian rodents. Studies in transgenic mice showed that collateral connections across MSNs of direct and indirect pathway are reduced by dopamine depletion (Taverna et al., 2008), while fast-spiking GABAergic striatal interneurons selectively increase the number of connections established with indirect-pathway MSNs (Gittis et al., 2011). These changes could enhance the degree of synchronization among striato-pallidal MSNs, and could induce alterations in the activity of the target regions. These plastic changes, however, have not yet been described in primates.

### GPe

The GPe strongly influences the activity of almost all of the other basal ganglia structures. Early studies in MPTP-treated monkeys reported that the average firing rate of GPe neurons was lower in MPTP-treated monkeys than in normal animals (Miller and DeLong, 1987, 1988). This observation has been corroborated in primate and rodent models of PD (Figure 3, Pan and Walters, 1988; Filion and Tremblay, 1991; Heimer et al., 2002; Soares et al., 2004; Mallet et al., 2008a). Recordings in PD patients found that the firing rates of GPe neurons were similar to that found in MPTP-treated monkeys (Hutchison et al., 1994; Sterio et al., 1994).

The reduced activity of GPe neurons after dopaminergic depletion could result from an increased activity of indirect pathway MSNs, as the “rate” model would predict (Figure 1, “Parkinsonism”). This view has also been incorporated into some of the models of basal ganglia dysfunction in the parkinsonian state (Terman et al., 2002). An alternative possibility is that reduced pallidal activity could result from a loss of autonomous pace making activity of these neurons after dopamine depletion. This idea is supported by studies in rodent models of parkinsonism in which a proportion of GPe neurons was found to show reduced spontaneous activity (Chan et al., 2010). In these studies, the number of “silent” GPe neurons correlated with the severity of the motor deficits.

In addition to the decreased firing rate, the incidence of burst discharges is increased in GPe neurons, as reported in MPTP-treated monkeys (Soares et al., 2004; Wichmann and Soares, 2006) and 6-OHDA treated rodents (Vila et al., 2000; Ni et al., 2001; Breit et al., 2007). In PD patients, there was a high degree of bursting in GPe neurons, reminiscent of that seen in MPTP-treated monkeys (Hutchison et al., 1994; Magnin et al., 2000). Also, individual neurons with oscillations in the (approximately) 3–8 and 8–15 Hz frequency bands are more frequently found in GPe of MPTP-treated than in normal monkeys (Raz et al., 2000; Soares et al., 2004). A similar observation has been made in the GP of 6-OHDA treated rats (Magill et al., 2001).

Furthermore, the normally uncorrelated activity of neighboring GPe neurons (Nini et al., 1995; Bar-Gad et al., 2003) becomes more synchronized after MPTP treatment (Nini et al., 1995; Raz et al., 2000; Heimer et al., 2002; Morris et al., 2005). The parkinsonian state is similarly associated with increased synchrony of neuronal activity in the pallidum in rodents (see below), as well as in most of the other basal ganglia structures. While the cause of the increased synchrony remains under investigation, it appears to be a pervasive feature of basal ganglia activity in parkinsonism that may contribute (or even underlie) other firing abnormalities, such as the appearance of oscillatory activity patterns or the finding of less specific sensory responses of neurons in the motor circuit (Rothblat and Schneider, 1995; Schneider and Rothblat, 1996; Boraud et al., 2000; Cho et al., 2002; Prokopenko et al., 2004).

Several recent rodent studies have demonstrated considerable heterogeneity among GPe neurons, suggesting distinctive patterns of projections, protein expression and electrophysiological activity (Sato et al., 2000; Kita, 2007; Mallet et al., 2008a, 2012; Mastro et al., 2014). Studies in rats have defined two major GPe neuronal populations with opposing phase relationships with cortical oscillatory activity, so-called prototypic and arkypallidal cells (Zold et al., 2007a,b; Mallet et al., 2008a, 2012). Compared with controls, the level of synchrony of firing between prototypic or arkypallidal cells, and between each of these groups and cortical activity is increased after 6-OHDA treatment (Mallet et al., 2008a; Zold et al., 2012). It has recently been proposed that these two types of GPe neurons, and the connections they establish among themselves and with the rest of the basal ganglia, may have an important role in the development of the pathological oscillations in parkinsonism (Nevado-Holgado et al., 2014). It is not (yet) clear whether these rodent findings can be generalized to primates. In the early reports of primate GPe electrophysiological activities, DeLong described neurons with high-frequency firing rates interspersed by pauses, and a second, less frequently encountered, type of neurons with low frequency discharge and bursts (DeLong, 1971). It has been suggested that these neuronal types might correspond, respectively to the prototypic and arkypallidal types described in rodents (Mallet et al., 2012), but the parallel remains unclear.

As in the striatum, dopamine depletion appears to be associated with morphologic and functional changes in connectivity in GPe. Thus, intrapallidal collaterals, which seem to exert only weak inhibition among neighboring neurons under normal conditions (Bar-Gad et al., 2003) appear to be functionally strengthened in 6-OHDA treated rats (Miguelez et al., 2012; Nevado-Holgado et al., 2014), and there is an increase in the abundance and strength of synaptic contacts between GPe and STN after dopaminergic depletion (Fan et al., 2012). It is not clear how these plastic changes are triggered. They may be an adaptive response, acting to reduce excessive STN activity (see below), at the price of reinforcing aberrant interneuronal synchronization.

### STN

Based on the initial finding that the mean firing rate of STN neurons is increased after MPTP-treatment in monkeys (Miller and DeLong, 1987; Bergman et al., 1994), later corroborated in several primate and rodent studies (Hassani et al., 1996; Bezard et al., 1999; Vila et al., 2000; Magill et al., 2001; Soares et al., 2004), most authors agree that PD is associated with excessive activity in the STN. This concept has strongly influenced the development of models of changes in the basal ganglia thalamocortical circuit in parkinsonism, and the development of surgical strategies for PD (DeLong and Wichmann, 2012).

While increased firing of STN neurons is considered a hallmark of the pathological electrical activities in parkinsonism, other changes in firing activities in the STN may be similarly important. For instance, similar to pallidal neurons, STN neurons show increased bursting, in parkinsonian animals, and probably also in parkinsonian patients (Figure 3, Bergman et al., 1994; Levy et al., 2000; Soares et al., 2004; Wichmann and Soares, 2006; Steigerwald et al., 2008; Tachibana et al., 2011). Bursting-related measures, such as the intra-burst firing rate of neurons, are among the most discriminative features of parkinsonism in parkinsonian monkeys (Sanders et al., 2013) and correlate strongly with the severity of parkinsonism in patients with PD (Sharott et al., 2014).

The proportion of neurons in STN with oscillatory firing is also markedly increased in the parkinsonian state, particularly in the 3–8 and 8–15 Hz frequency ranges in MPTP-treated monkeys (Bergman et al., 1994; Soares et al., 2004; Rivlin-Etzion et al., 2006; Moran et al., 2012; Galvan et al., 2014). These oscillations are coherent with those of neurons that are simultaneously recorded in the STN targets, GPe and GPi (Moran et al., 2012). In PD patients, many STN neurons also show oscillations, but at higher (15–30 Hz) frequencies (Levy et al., 2002b; Weinberger et al., 2006). Neurons with oscillatory activity are more likely to exhibit, in addition, an increase in tonic firing (Bergman et al., 1994; Deffains et al., 2014). Studies in humans have also shown that STN neurons in PD patients with limb tremor tend to show synchronized firing (Levy et al., 2000).

Numerous studies have analyzed the characteristics of LFP signals recorded from the STN area in patients with advanced parkinsonism. Such recordings can conveniently be made, using perioperative recordings in patients who undergo therapeutic DBS lead placement procedures that target the STN, using the implanted DBS electrodes as recording electrodes. It is worth noting that the (usually bipolar) “LFP” recordings in these cases refer to potentials between two contacts of the DBS electrode which are at least 1.5 mm apart, thus reflecting potential differences between separate groups of neurons. It is not clear how such potentials relate to the membrane potential fluctuations in small groups of neurons that are usually recorded as LFPs in animal experimentation. These studies have shown that the disease is associated with prominent oscillations in the STN in the beta band (for review, see Stein and Bar-Gad, 2013) which can be reduced by dopaminergic medications and high-frequency stimulation of the STN (Brown et al., 2001; Levy et al., 2002a; Priori et al., 2004; Wingeier et al., 2006; Kühn et al., 2008b; Bronte-Stewart et al., 2009). Some of the studies in 6-OHDA treated rats have also shown increased beta band power in STN LFPs, as compared with normal animals (Sharott et al., 2005a).

However, beta-band oscillations in LFP recordings from MPTP-treated monkeys are not as prominent as they are in humans (despite the presence of a clearly recognizable parkinsonism, Devergnas et al., 2014). Explanations for the discrepancy between these studies and those in human patients include the possibility that the degree of synchrony in the parkinsonian monkeys may not be sufficient to lead to recordable LFP oscillations or that the recording conditions are substantially different. Another important consideration is that the main frequency of normal and abnormal oscillations in these animals may differ from that in humans. It has been suggested that the 8–15 Hz frequency range in monkeys may be equivalent to the beta band in humans (Stein and Bar-Gad, 2013).

The interactions between the reciprocally connected GPe and STN (Smith et al., 1998) may be particularly important in the development of bursts and oscillations in these two structures (Plenz and Kitai, 1999; Ni et al., 2000a; Cruz et al., 2011). Modeling studies have proposed that the STN-GPe connections may generate rhythmic or irregular patterns of activity. Increased input from indirect pathway MSNs onto GPe neurons tends to promote rhythmicity and abolish irregular firing (Terman et al., 2002). After dopamine depletion, the pallidosubthalamic circuits are strengthened by an increase in the number of synaptic connections between GPe terminals and STN, as has been demonstrated in 6-OHDA treated rodents (Fan et al., 2012). Such increased inhibition from the GPe may promote hyperpolarization-induced rebound bursting of STN cells (Beurrier et al., 1999; Bevan et al., 2007), and account for changes in the temporal structures of burst discharges in pallidum and STN in MPTP-treated monkeys (Wichmann and Soares, 2006). Furthermore, studies on parkinsonian rats have suggested that oscillations that originate in striatum, cortex or thalamus may be amplified in the GPe-STN network which would act as a non-linear oscillator with self-adjusting resonance frequencies (Nevado-Holgado et al., 2014).

Rhythmic activity in the STN is closely related to that in cortex (Magill et al., 2000), and this correlation is exacerbated after dopamine depletion in 6-OHDA treated rats (Magill et al., 2001), with a peak coherent activity between cortex and STN in the beta range (Sharott et al., 2005b). Similarly correlated cortex-STN activity has been described to be dopamine-dependent in PD patients (Williams et al., 2002; Shimamoto et al., 2013).

### GPi and SNr

As could be predicted by the increased firing of STN neurons and the reduced firing of GPe neurons, the firing rates of GPi and SNr neurons are increased after dopamine depletion in most studies of parkinsonian primates (Figure 3, Miller and DeLong, 1987, 1988; Filion and Tremblay, 1991; Wichmann et al., 1999; Heimer et al., 2002; Soares et al., 2004). In PD patients, the average firing rate of GPi neurons was similar to that seen in MPTP-treated monkeys (Hutchison et al., 1994; Sterio et al., 1994), and it was higher than the average discharge rate in dystonia patients (Starr et al., 2005).

Compared to normal animals, in the GPi of MPTP-treated monkeys, there was a smaller proportion of neurons that decreased activity during movement onset (Leblois et al., 2006). Instead, the authors found that most GPi neurons showed increased activity around the time of movement. (Leblois et al., 2006). This abnormal movement-related excitation of GPi neurons could result in excessive inhibition of thalamic neurons (and thus, help explain bradykinesia during voluntary movements in parkinsonism).

GPi neurons of MPTP-treated monkeys also show increased oscillations and bursting activities (Raz et al., 2000; Soares et al., 2004; Leblois et al., 2007). Similarly, in PD patients burst firing is found in both oscillatory and non-oscillatory GPi cells (Chan et al., 2011). As found in GPe, synchronized activity among GPi neurons is more prominent after MPTP treatment in monkeys (Nini et al., 1995; Bergman et al., 1998; Raz et al., 2000; Morris et al., 2005; Leblois et al., 2007) and in PD patients (Hurtado et al., 1999; Levy et al., 2000). In studies of LFP signals, recorded from DBS electrodes in GPi patients (Silberstein et al., 2003; Weinberger et al., 2006), the relative power in the beta band (11–30 Hz) was higher in parkinsonian patients than in those with dystonia, while the power in the 4–10 Hz range was higher in the dystonic group (Silberstein et al., 2003). In MPTP-treated monkeys, however, the oscillatory activities are predominant at lower frequencies (7.8–15.5 Hz, Devergnas et al., 2014).

Oscillatory activities can also be identified in the SNr in 6-OHDA treated rats. Recordings using chronically placed electrodes in the SNr of unilaterally 6-OHDA lesioned animals showed increased oscillatory LFP activity and increased synchronization (entrainment) of single cell activity to LFP oscillations in the 12–40 Hz range in the dopamine-depleted hemisphere compared to the non-lesioned side (Avila et al., 2010; Brazhnik et al., 2012, 2014). Brazhnik et al. found that “active” states, such as grooming and walking, were related to relatively higher frequencies, while rest and REM sleep were associated with lower frequencies (Brazhnik et al., 2014).

While it is not clear how such oscillations are generated or modulated, the finding of high coherence of oscillations among STN, GPe and GPi in parkinsonian individuals (Moran et al., 2012) suggests that the oscillations in the basal ganglia output nuclei may be related to oscillations in the other nuclei. For instance, in PD patients, low frequency stimulation of the STN enhances the synchronization at similar frequencies in the GPi, while higher frequency stimulation of STN suppresses the beta-range oscillations in GPi (Brown et al., 2004). It is conceivable that the altered balance between direct and indirect pathways in the parkinsonian state (Mallet et al., 2006) may allow (oscillatory) STN output to strongly “drive” GPi neurons.

There is evidence that, after dopaminergic depletion, there is a disruption of the somatory-sensory representation in the GPi, as indicated by a reduction in the specificity of responses to sensory stimuli in the entopeduncular nucleus of MPTP-treated cats (Rothblat and Schneider, 1995) and an increased number of GPi neurons that respond to movement in monkeys (Leblois et al., 2006).

### Thalamus

Studies in MPTP-treated (parkinsonian) monkeys suggest that metabolic activity is increased in VA and VL (Mitchell et al., 1989; Rolland et al., 2007), perhaps reflecting increased basal ganglia input. The downstream effects of abnormal basal ganglia output on firing rates in thalamus have been studied to a limited extent and comparative studies of neuronal activity in the basal ganglia-receiving regions of the thalamus have been inconsistent. While some studies show decreased neuronal firing (Vitek et al., 1994; Schneider and Rothblat, 1996; Ni et al., 2000b; Kammermeier et al., 2014), others found no firing rate change (Pessiglione et al., 2005), and others show increase in firing rates (Bosch-Bouju et al., 2014). In PD patients, neurons in the basal ganglia-receiving areas of thalamus have a reduced mean firing rate, compared to similar recordings from non-PD patients (Molnar et al., 2005; Chen et al., 2010).

Recent studies in rats have shown that, under normal conditions, the firing rate of neurons in the motor thalamus is modulated during reaching movements, and that the responsiveness of thalamic cells during such movements is greatly reduced in 6-OHDA lesioned (Bosch-Bouju et al., 2014). Such task-related changes in thalamic activity could contribute to some of the deficits in motor performance in parkinsonism.

Studies in MPTP-treated monkeys have suggested that burst discharges are increased in the motor thalamus (Guehl et al., 2003; Pessiglione et al., 2005) and a similarly high incidence of burst firing has been found in corresponding areas in PD patients (Zirh et al., 1998; Magnin et al., 2000; Molnar et al., 2005). The thalamic bursts were shown to fulfill the criteria of “rebound” bursts (Magnin et al., 2000), suggesting that the bursts could result from hyperpolarization of thalamic neurons driven by increased inhibitory basal ganglia input via a T-type calcium channel dependent mechanism. These results may, however, not only relate to the changed motoric state of the subject, but also to parkinsonism-related changes in the state of arousal. Indeed, patients and animals tend to be less awake in the parkinsonian state, which, independent of other factors, may contribute to the finding of a higher incidence of burst discharges in the thalamus.

Reports of the effects of parkinsonism on oscillatory activity in the motor thalamus have been inconsistent. LFP recordings from the pallidal receiving area of PD patients with drug-resistant tremor showed prominent oscillations in the tremor frequency range (4–9 Hz), strongly correlated with oscillations recorded in frontal cortex (Sarnthein and Jeanmonod, 2007). Our recent findings on oscillatory activity patterns in parkinsonian monkeys suggest that single-cell oscillatory activities increase in the 3–13 Hz range of frequencies, and are mildly reduced in the gamma-range of frequencies in the basal ganglia-receiving area of the thalamus (Kammermeier et al., 2014).

The disruption in somatosensory representation is also apparent in the motor thalamus. The sensory responses of VA/VL neurons were found to be less specific in MPTP-treated monkeys than under normal conditions (Pessiglione et al., 2005). In the thalamus of these animals, the spiking activity of neighboring VA/VL neurons was more frequently correlated than under normal conditions (Pessiglione et al., 2005).

Several of the alterations in firing described in the basal ganglia-receiving portion of the thalamus in parkinsonism, such as increased bursting and abnormal sensory processing, have also been described for the thalamic regions that receive cerebellar inputs (Guehl et al., 2003; Molnar et al., 2005; Pessiglione et al., 2005; Chen et al., 2010). In fact, a recent study of single unit activity in the cerebellar and basal ganglia-receiving regions of the thalamus in PD patients, found that oscillations of single neurons in the beta range were prominent in the cerebellar regions of the thalamus, but not in the basal ganglia area (Basha et al., 2014). The recently described interconnections between the cerebellum and the basal ganglia (Bostan et al., 2010, 2013; Bostan and Strick, 2010) could have a role in the development of electrophysiological alterations and the parkinsonian motor deficits (Lewis et al., 2013), but it is also possible that such oscillations reflect changes in cortical oscillatory patterns which may then be fed back to the thalamus via corticothalamic projections that modulate the excitability of thalamocortical neurons. It is important to note that interventions aimed at the cerebellar receiving thalamus (lesioning or stimulation) are an established treatment for parkinsonian tremor, suggesting that the cerebello-thalamo-cortical pathway may be involved in the generation of parkinsonian tremor (Benabid et al., 1991; Schuurman et al., 2000).

Besides the VA/VL, the CM/PF nuclei also receive inputs from the basal ganglia, and then send a massive projection back to the striatum (Smith et al., 2014a). The impact of CM/PF activity on basal ganglia functions under normal conditions is just starting to emerge (Smith et al., 2011), and remains almost unexplored in the parkinsonian state. In 6-OHDA treated rats, recordings in the PF (the rodent equivalent of the primate CM, Smith et al., 2004) there is a reduction in the number of neurons with spontaneous activity, a large proportion of cells develop oscillations at 0.3–2.5 Hz, and there is a reduction in low threshold spike (LTS) bursts (Parr-Brownlie et al., 2009). The changes in PF neurons, however, do not seem to be driven by changes in the basal ganglia (Parr-Brownlie et al., 2009). LFP recordings in the CM in PD patients described an increase in gamma band activity after levodopa treatment and a decrease in beta-band activity, but the latter was seen in only one of three patients (Kempf et al., 2009). CM/PF activity changes may not only be shaped by altered inputs from the basal ganglia, but also by the fact that CM and PF neurons degenerate early in parkinsonism, as reported for PD patients (Henderson et al., 2000a,b), and MPTP-treated monkeys (Villalba et al., 2014). It is unclear how the loss of CM/PF neurons affects the activity of the remaining cells in the CM/PF complex or in other basal ganglia structures that receive afferents from it.

### Cortex

Early studies of neuronal activity of cortical neurons in primary motor (M1) or supplementary cortices in monkeys reported that the spontaneous activity of these neurons did not change with MPTP treatment, but that movement-related discharges were reduced in parkinsonian animals (Doudet et al., 1990; Watts and Mandir, 1992; Goldberg et al., 2002; Escola et al., 2003). A later study, in which M1 projection neurons were identified in primates on the basis of responses to antidromic stimulation, demonstrated that the observed reductions in firing rates were restricted to neurons projecting to the pyramidal tract, but did not affect those projecting to the striatum (Pasquereau and Turner, 2011). This suggests that the pyramidal tract projecting subpopulation of cortical neurons might be particularly involved in the expression of motor problems associated with parkinsonism.

In M1 of MPTP-treated monkeys, the number of bursting neurons is increased compared to normal animals (Goldberg et al., 2002). As reported for the changes in firing rates, corticospinal, but not corticostriatal, M1 neurons showed increased bursting after MPTP-treatment (Pasquereau and Turner, 2011). Increased neuronal synchrony has also been found among M1 neurons in MPTP treated monkeys (Goldberg et al., 2002).

In addition to the aforementioned single neuron recording studies, there is a large number of studies of cortical field potential oscillations, mostly using EEG and ECoG recordings. It is noteworthy that EEG signals are the only electrophysiologic signals from human patients for which true control data (from normal individuals) are available. The amplitude of these signals tends to be larger and more robust than similarly recorded LFP signals recorded from the basal ganglia because of the laminar organization of cortex that contrasts with the non-laminar architecture of the basal ganglia. Studies of EEG recordings have found abnormally large delta (1–3 Hz) and theta band (4–7 Hz) activities in parkinsonian patients at rest, both in M1 (Soikkeli et al., 1991; Neufeld et al., 1994; Serizawa et al., 2008) and in other cortical regions (Silberstein et al., 2005; Babiloni et al., 2011; Morita et al., 2011). Animal studies have shown an increase of beta band oscillations in ECoG signals recorded in the frontal cortex of 6-OHDA treated rats (Sharott et al., 2005b; Brazhnik et al., 2012), and an increase of low frequency oscillations (<15.5 Hz) in M1 of MPTP treated monkeys (Devergnas et al., 2014).

These cortical activity changes could be secondary to the aberrant activity in the basal ganglia and thalamus, but could also originate in the cerebral cortex itself, perhaps reflecting the effects of cortical dopamine depletion (Lindenbach and Bishop, 2013). In addition, the effects of the loss of other neurotransmitters (such as noradrenaline, acetylcholine and serotonin) (Gaspar et al., 1991; Braak et al., 2003) and the neuronal degeneration in the motor cortex in PD (MacDonald and Halliday, 2002) remain to be further studied.

## Role of dopamine in development of electrophysiological changes in parkinsonism

Although it is often mentioned that the changes in electrophysiologic activity that were discussed above are consequent to the loss of dopamine in the striatum, the link between the disrupted striatal dopaminergic transmission and altered activity patterns in basal ganglia, thalamus and cortex is not entirely clear. Several studies have attempted to investigate this issues using locally or systemically administered dopamine receptor agonists or antagonists.

In PD patients, systemic infusions of therapeutic effective doses of the non-selective dopamine agonist apomorphine was shown to decrease the firing rates of GPi cells, and of STN cells with activity related to limb tremor; it also decreased the proportion of STN and GPi cells with responses to passive movements (Levy et al., 2001). However, in other studies, dopamine receptor agonist treatment did not fully reduce burst firing in basal ganglia neurons of parkinsonian animals or patients (Tseng et al., 2000; Lee et al., 2001; Levy et al., 2001), and was shown to even promote neuronal bursting when locally applied in the STN (Baufreton et al., 2003; Galvan et al., 2014) or in GPi or SNr (Kliem et al., 2007). Furthermore, acute blockade of dopaminergic transmission failed to increase beta band activity in subthalamic LFPs of rats, despite the presence of motor symptoms (Mallet et al., 2008b).

While increased bursting in the parkinsonian state may not fully respond to dopamine replacement, the increased synchrony found among striatal MSNs, or between MSNs and GP neurons appears to be more tightly controlled by the presence or absence of dopamine (Heimer et al., 2002; Burkhardt et al., 2007), suggesting that the asynchronous firing of basal ganglia neurons in the normal state is an actively maintained state. Details of the mechanism and extent of this identified decorrelating function of dopamine are lacking.

The effects of dopamine on oscillatory activity patterns have also been examined. Increased oscillations in the SNr in the 25–40 Hz become prominent after complete, but not partial, 6-OHDA lesions, suggesting that an extensive lesion of the dopaminergic system is a prerequisite for the appearance of increased oscillatory activity (Quiroga-Varela et al., 2013). Importantly, motor impairments are evident at early stages of the dopaminergic degeneration processes. Thus, the increased oscillatory activity may not be an indicator (or cause) of early parkinsonism.

Systemic administration of dopaminergic agents reduces the degree of beta band oscillations in LFP recordings from the STN and GP in parkinsonian patients (Brown et al., 2001; Cassidy et al., 2002; Levy et al., 2002a; Priori et al., 2004; Kühn et al., 2008a; Bronte-Stewart et al., 2009), and from the SNr in 6-OHDA treated rats (Brazhnik et al., 2014). These agents also restore, to some extent, EEG abnormalities in M1 in MPTP-treated monkeys (Devergnas et al., 2014). Interestingly, in the latter study, it was observed that beta-band activity in the STN was reduced even in the absence of primary pathologic oscillations, suggesting that levodopa may not specifically normalize pathologic oscillations in the parkinsonian state, but may more generally suppress beta-band oscillations.

As already mentioned, the lack of dopamine is (directly or indirectly) accompanied by long-term morphological (and potentially functional) changes at different levels of the basal ganglia-thalamo-cortical circuits that may contribute to the abnormal electrophysiologic activities. These morphological changes are not necessarily a direct consequence of the dopaminergic loss, and may therefore escape the regulation of acute dopaminergic interventions.

## Relationship between the different pattern abnormalities and the behavioral manifestations of parkinsonism

There is little question that neuronal activities in the basal ganglia-thalamo-cortical circuits are altered in PD, but it is less clear to what extent these alterations account for the motor (or non-motor) symptoms of the disease.

One experimental approach to this problem has been to examine the effects of inactivation of the dysfunctional brain region(s). Experimental and clinical evidence indicate that lesions or inactivation of the “overactive” STN or GPi ameliorate parkinsonian symptoms in parkinsonian animals and in PD patients (Bergman et al., 1990; Aziz et al., 1991; Guridi et al., 1994; Wichmann et al., 1994; Parkin et al., 2001; Alvarez et al., 2005; Coban et al., 2009; Yoon et al., 2014). The knowledge gained from such lesioning experiments is limited, however. One problem is that lesions disrupt all activity in the lesioned nucleus, thus providing little insight into the role of specific activity changes. A more general consideration is that improvement of a behavioral abnormality after a lesion does not necessarily confirm that activity in the lesioned brain region was a primary cause for the abnormal behavioral patterns. For instance, the lesion could have induced plastic changes in other brain regions that could be responsible for the behavioral improvement.

**Figure 4:**
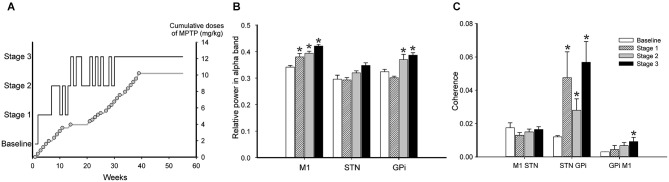
**Relationship between oscillatory activity changes in the cortico-basal ganglia network and progression of parkinsonism, in an MPTP-treated monkey**. The animal was chronically instrumented for recording of EcoG signals from the primary motor cortex (M1). In addition, local field potential signals were recorded from STN and GPi through macroelectrodes that were repeatedly inserted into the same basal ganglia locations throughout the course of the experiment. **(A)** Temporal progression of the development of parkinsonism induced by MPTP injections, based on weekly evaluations. The solid black line shows the stage of parkinsonism. The cumulative dose of MPTP is shown as a gray line with open circles, where each circle corresponds to a single MPTP injection. Stages 1–3 represent mild, moderate, and moderately-severe parkinsonism, as judged by a parkinsonian rating scale. **(B)** Relative spectral power (mean ± SEM) of M1, STN and GPi signals (EEG, LFPs) across the different stages. **(C)** Coherence between M1 and STN, STN and GPi, and M1 and GPi signals in the 7.8–15.5 Hz frequency range across different stages of parkinsonism during periods of wakefulness. *p < 0.05 vs. baseline (2-way ANOVA with *post hoc* Holm-Sidak test). Modified from Devergnas et al. (2014), with permission.

An alternative way to study the possible link(s) between the basal ganglia abnormalities and parkinsonism is to examine the temporal relationship between the electrophysiologic changes and the behavioral state. An early study in monkeys in which repeated injections of small doses of MPTP resulted in the gradual development of parkinsonian signs suggested that changes in single cell firing in STN and GPi may precede the onset of motor signs (Bezard et al., 1999). This report contrasts, however with a more recent report which found activity changes in GPi only after the development of parkinsonian motor signs (Leblois et al., 2007). A related study by Sharott et al. (2014) found that the beta-band power of single- or multiunit activities recorded in the STN of parkinsonian patients was correlated with the severity of bradykinesia, and that sub-beta (<2–13 Hz) oscillations had a positive correlation with bradykinesia and axial symptoms. Measures of burst intensity (e.g., the intra-burst firing rate) were also correlated with parkinsonian signs (Sharott et al., 2014). Interestingly, the severity of tremor did not correlated with any of the measured abnormalities. In our own recent study of parkinsonism-related changes in LFP activities in the basal ganglia of chronically MPTP-treated monkeys, we did not find a correlation between oscillatory activity in the STN and motor impairments, but found instead that the severity of the motor signs was correlated with an increase in LFP power in low frequency bands (7.8–15.5 Hz) and a decrease in higher-frequency bands (23.4 Hz) in M1 and GPi. We also found an increase in coherence between M1 and basal ganglia oscillations in the 7.8–23.3 Hz frequency range to be associated with the severity of parkinsonism (Figure 4, Devergnas et al., 2014). Thus, it remains unclear if the early motor parkinsonian signs appear before the electrophysiological alterations can be detected (Quiroga-Varela et al., 2013).

Another approach to the possible link between neuronal activity changes and the motor signs is to analyze whether effective antiparkinsonian treatments are accompanied by “normalization” of activity patterns in the basal ganglia-thalamocortical circuit. Many studies have provided evidence that antiparkinsonian treatments reduce burst discharges (Filion et al., 1991; Shi et al., 2006; Xu et al., 2008), beta band oscillations (Brown et al., 2001; Levy et al., 2002a; Priori et al., 2004; Wingeier et al., 2006; Kühn et al., 2008b; Bronte-Stewart et al., 2009; Brazhnik et al., 2014) and abnormal synchronization within and among basal ganglia nuclei (Levy et al., 2000; Heimer et al., 2002; Hammond et al., 2007). Similarly, cortical coherence in the 10–35 Hz range, associated with the severity of parkinsonism, is reduced with STN-DBS (Silberstein et al., 2005). DBS in the STN also reduces neuronal entropy (a measure of the “disorder” of neuronal firing) in the GPe, GPi and motor thalamus (Dorval et al., 2008). These correlations obviously do not prove causality, however. Indeed, other studies have reported that these treatments do not always completely restore normal electrical activity in basal ganglia neurons (Lee et al., 2001; Levy et al., 2001; Heimer et al., 2006; Hahn et al., 2008; McCairn and Turner, 2009), or, as already mentioned, that the anti-oscillatory properties of dopaminergic treatments may be non-specific (Devergnas et al., 2014).

Finally, it has been studied whether parkinsonian signs can be induced or worsened by experimentally imposing “pathological” frequencies or patterns on the basal ganglia circuitry. As in other studies described above, these studies have not been fully conclusive. Thus, while low-frequency stimulation of the STN, presumably inducing beta-band oscillations, was described to worsen akinesia (Timmermann et al., 2004), other human and animal studies have found subtle or no effects of this intervention (Chen et al., 2007, 2011; Eusebio et al., 2008; Syed et al., 2012). As an alternatively experimental approach to induce parkinsonian symptoms, Soares et al. (2004) used ibotenic acid to lesion the GPe in normal monkeys, with the rationale to mimic the proposed lack of GPe activity in PD (Figure 1, “Parkinsonism”). Such GPe inactivation increased the firing of STN and GPi neurons, but did not result in development of parkinsonian motor signs (Soares et al., 2004).

Overall, despite significant experimental study, it remains unclear whether the changes found in the neuronal signals in the parkinsonian state can be clearly linked with the emergence of parkinsonism. It is important to remember that the electrophysiological alterations could be more prominent during specific phases or types of movements (Brazhnik et al., 2012, 2014; Lemaire et al., 2012; Quiroga-Varela et al., 2013; Bosch-Bouju et al., 2014). It also remains to be further clarified which electrophysiological activity changes are most specific for PD or its specific signs and symptoms (Sanders et al., 2013; but see Sharott et al., 2014). Studies of this topic in advanced human patients are obviously complicated by the fact that long-term exposure to antiparkinsonian treatments and non-dopaminergic changes may play significant (and poorly defined) roles.

## Use of the insights from pathophysiology studies to improve surgical PD therapies

While a causal link between the electrophysiologic abnormalities and parkinsonian motor signs remains elusive, our knowledge of the electrophysiological activities in the basal ganglia and related structures in PD can, nevertheless, be used to improve surgical therapies, such as DBS or lesioning approaches. For example, it may be possible to use the power distribution in LFP signals as a substitute for the currently used time-consuming microelectrode recording methods to guide the placement of DBS electrodes. In studies of intra-operatively recorded LFPs from DBS electrodes that were advanced towards the STN (Chen et al., 2006; Miyagi et al., 2009), the beta band power in LFP signals was found to be maximal at the surgical target in the dorsal STN (Chen et al., 2006; Miyagi et al., 2009). However, this technique is at present clearly limited by the low spatial resolution of LFP signal recordings (Zaidel et al., 2010). A related use of recordings from DBS electrodes is to choose the best stimulation contact of already implanted electrodes based on the amount of beta-band power that can be recorded from them (Ince et al., 2010).

Another application of our electrophysiologic knowledge is the use of disease-related electrophysiological characteristics to regulate DBS parameters in “closed-loop” or “adaptive” feedback regimes that dynamically adapt the therapy to fluctuations of disease severity, and may help to prolong the battery life of the implanted devices by cutting the duty-time of the implanted pulse generator (for review, see Priori et al., 2013). The potential success of adaptive DBS for PD has been computationally modeled (Santaniello et al., 2011), and experimentally demonstrated in studies that used cortical single neuron activity patterns to trigger STN stimulation in parkinsonian monkeys (Rosin et al., 2011). Of course, in clinical use, LFP-based methods of adaptive DBS would be more practical. The first attempt to use LFP characteristics to control stimulation parameters was recently published, using short-term changes in the beta-band power of STN LFP signals to trigger STN-DBS in PD patients (Little et al., 2012, 2013). In these experiments, the adaptive DBS was at least as effective as continuous DBS. Interestingly, the duty cycle of closed loop-controlled DBS progressively shortened in these studies, even over relatively short stimulation periods, suggesting that plastic changes could be triggered by this type of intermittent STN stimulation that may eventually make STN DBS less and less necessary to control the parkinsonian signs in a given patients. The signal analysis and control of the DBS device still required an external computer and the miniaturization and overall power consumption of an implantable signal sampling, processing and stimulation system remain important limitations (Starr and Ostrem, 2013). Other approaches have also been proposed, including specifically the use of measures of cortical phase-amplitude coupling between beta- and gamma-band ECoGs signals to trigger DBS (de Hemptinne et al., 2013), or the use of “coordinated reset” stimulation, a stimulation regime based on computer modeling, designed to minimize pathologic synchronization of activity patterns in the STN and related nuclei (Tass et al., 2012; Adamchic et al., 2014).

## Conclusions

The literature review presented above shows that similar changes in neuronal activities (changes in firing rates, increases in bursting, synchrony and oscillatory activities in the beta range) have been seen in almost all basal ganglia nuclei, both in PD patients and in animal models of the disease (Table 1). In fact, PD remains the only neurologic disease for which this level of detailed information regarding specific network alterations is available. At the most general level, the described changes jointly paint the picture of an altered network state that is disruptive for motoric and non-motoric functions.

Despite all of the progress that has been made in this field, there is certainly much need for further investigations of the role of specific circuit nodes or interactions between them in producing or relaying the described abnormalities, and of the link(s) between these changes and the behavioral signs of the disease. There is clearly also a need for further studies of the cellular and molecular underpinnings of these changes. In addition, experimental studies need to pay close attention to the species differences in the basal ganglia circuits among rodents (and other smaller mammals) and non-human primates and patients (Parent, 1986; Smith et al., 2014b).

The potential translational payoff of the accumulated knowledge of activity changes in the parkinsonian state is very large. Insights gained from these studies may help us to develop some of these changes (such as EEG changes) into early biomarkers for treatment trials in patients, and there are already many trials underway to use them to guide neuromodulation therapies such as DBS. Furthermore, knowing how specifically neuronal activity patterns changes in the parkinsonian state may help us to develop treatments that specifically address these activity pattern changes rather than simply replace dopamine as is currently done. Combined, these efforts promise to translate into more specific therapies for patients with PD that are both, more effective and less encumbered by adverse effects than the currently available treatments.

## Conflict of interest statement

The authors declare that the research was conducted in the absence of any commercial or financial relationships that could be construed as a potential conflict of interest.
